# Physician Burnout in Primary Care during the COVID-19 Pandemic: A Cross-Sectional Study in Portugal

**DOI:** 10.1177/21501327211008437

**Published:** 2021-04-12

**Authors:** Sofia Baptista, Andreia Teixeira, Luísa Castro, Maria Cunha, Carla Serrão, Andreia Rodrigues, Ivone Duarte

**Affiliations:** 1 Department of Community Medicine, Information and Health Decision Sciences (MEDCIDS), Faculty of Medicine, University of Porto, Porto, Portugal; 2 Center for Health Technology and Services Research (CINTESIS), University of Porto, Porto, Portugal; 3 Foz do Douro Health Center, ACeS Porto Ocidental, Porto, Portugal; 4 Instituto Politécnico de Viana do Castelo, Viana do Castelo, Portugal; 5 School of Health of the Polytechnic of Porto, Porto, Portugal; 6 Arte Nova Family Health Center, Aveiro, Portugal; 7 School of Education of the Polytechnic of Porto, Porto, Portugal; 8 Center for Research and Innovation in Education, Porto, Portugal; 9 Darque Health Center, Alto Minho Local Health Center, Viana do Castelo, Portugal

**Keywords:** burnout, primary care, COVID-19, pandemic, general practice

## Abstract

**Background:**

Primary care physicians have been present on the frontline during the ongoing pandemic, adding new tasks to already high workloads. Our aim was to evaluate burnout in primary care physicians during the COVID-19 pandemic, as well as associated contributing factors.

**Methods:**

Cross-sectional study with an online questionnaire disseminated through social media, applying the snowball technique. The target population was primary care physicians working in Portugal during the first outbreak of the COVID-19 pandemic. In addition to sociodemographic data, the questionnaire collected responses to the Copenhagen Burnout Inventory (CBI), the Resilience Scale and the Depression, Anxiety, and Stress Scales (DASS-21). Data were collected from May 9 to June 8, 2020, a period comprising the declaration of a national calamity and then state of emergency, and the subsequent ease of lockdown measures. Levels of burnout in 3 different dimensions (personal, work, and patient-related), resilience, stress, depression, and anxiety were assessed. Logistic regression analyses were conducted to identify factors associated with burnout levels.

**Results:**

Among the 214 physician respondents, burnout levels were high in the 3 dimensions. A strong association was found between gender, years of professional experience, depression and anxiety, and burnout levels.

**Conclusions:**

Physician burnout in primary care is high and has increased during the pandemic. More studies are needed in the long term to provide a comprehensive assessment of COVID-19’simpact on burnout levels and how to best approach and mitigate it during such unprecedented times.

## Background

The first confirmed case of COVID-19 in Portugal was reported on March 2, 2020. For Portuguese physicians, that day marked the beginning of a great challenge that would soon be declared a global pandemic by the World Health Organization (WHO).^1^

Burnout is defined in the 11th Revision of the International Classification of Diseases (ICD-11) as a syndrome resulting from chronic workplace stress that has not been successfully managed. It is characterized by 3 dimensions: feelings of energy depletion or exhaustion; increased mental distance from one’s job, or feelings of negativism or cynicism related to one’s job; and reduced professional efficacy.^2^ It is considered a relevant occupational health hazard among healthcare workers (HCW) and has a significant impact on professionals, patients and health institutions. Burnout was already a problem in Portugal prior to COVID-19, with 43.6% of Portuguese physicians reporting high levels of burnout.^3^

As the COVID-19 pandemic increases pressure on healthcare systems worldwide, physician burnout levels are also expected to rise. With a total of 32 500 confirmed cases and 1410 deaths from COVID-19 as of May 31,^4^ the pandemic has brought new stressors possibly contributing to physician burnout, such as fears of becoming infected or infecting a relative, lack of appropriate personal protective equipment, lack of access to up-to-date information and communication, limited time with family and friends, reductions in economic revenue, and increased demands from childcare and household tasks.^5-7^

Around the world, general practitioners and family doctors were on the frontline fighting the spread of infection. In the Portuguese health care system, the family doctor acts as a gatekeeper; while maintaining this role during the pandemic, they had to adjust and add new activities to their daily schedules (such as the diagnosis and monitoring of infected patients, prescribing their respective sick leaves, and adapting to the use of telemedicine for most consultations) while keeping up-to-date with new evidence and guidelines on the novel coronavirus, all in addition to their normal preventive and curative medical activity.^8-10^

In a study to understand the impact of COVID-19 around the world that included a total of 2707 participants from 60 countries, 51% of HCWs reported burnout.^11^ In a recent study of 2008 HCWs in Portugal during the pandemic, where burnout was measured by the validated Portuguese version of the Copenhagen Burnout Inventory (CBI),^12^ high burnout levels were reported by 52.5% of participants for personal burnout, 53.1% for work-related burnout, and 35.4% for patient-related burnout.^13^ Another work focusing on the levels of burnout in HCW in Italy during the COVID-19 pandemic used the Maslach Burnout Inventory (MBI), whose subscales assess levels of emotional exhaustion, depersonalization and personal accomplishment. This study revealed high levels of emotional exhaustion in 41% and high levels of depersonalization in 27%.^14^ Wu et al. specifically compared levels of burnout in frontline vs. other HCWs in Wuhan, China to find that the first had significantly lower levels of burnout and were less worried about becoming ill compared to those in the “usual ward” group,^15^ highlighting that all HCWs should be considered when designing well-being policies, irrespective of their position.

Kristensen et al. developed the CBI based in the concept that burnout is not just fatigue or exhaustion, it is the attribution of these feelings to specific domains of one’s life, as a result CBI is a tool with 3 subscales: personal, work, and client-related burnout. The personal burnout subscale measures feelings of physical, emotional, and mental fatigue and exhaustion. The work-related burnout subscale assesses the symptoms that respondents attribute to work. The client-related burnout subscale describes the aforementioned feelings that respondents attribute to their work with clients.^16^

To our knowledge, no study to date has focused on assessing primary care physicians’ burnout levels in addition to stress, depression, anxiety, and resilience during the COVID-19 outbreak, nor the associated contributing factors.

## Methods

### Study Design

Cross-sectional study with an online questionnaire disseminated through social networks, spread using the snowball technique.

### Participants

The study population was Portuguese-speaking primary care physicians working in Portugal during the COVID-19 pandemic.

### Data Collection and Variables

Data were collected from May 9 to June 8, 2020, a period comprising the declaration of a national calamity, the subsequent state of emergency, and the ease of lockdown measures that followed. The questionnaire, developed using Google^®^ Forms, was spread as a web link through social networks and institutional mailing lists. All participants gave their informed consent to participate in this study and anonymity was assured. The first section of the questionnaire addressed sociodemographic data. Psychological variables collected in the following sections included the Copenhagen Burnout Inventory (CBI), the Resilience Scale and the Depression, Anxiety, and Stress Scale (DASS-21).

The validated Portuguese version of the CBI was used to measure burnout.^12^ This is a 19-item scale with 3 subscales: personal (6 items), work (7 items), and patient-related burnout (6 items). Each item is answered on a 5-point Likert scale (always/to a very high degree = 100, often/to a high degree = 75, sometimes/somewhat = 50, seldom/to a low degree = 25, and never/almost never/to a very low degree = 0). The score for each subscale is the average of item scores within the subscale, ranging from 0 to 100. A score of 50 or higher in any of the subscales was considered high-level burnout.^12,16^ These subscales demonstrated high internal consistency (original version: α = .84; Portuguese version: α = .86, where α is the Cronbach’s alpha).^12,16^ In the current study, α was .91, .89, and .89 for personal burnout, work-related burnout, and patient-related burnout, respectively.

The Resilience Scale is composed of 25 items, each with a 7-point Likert response scale ranging from 1 (strongly disagree) to 7 (strongly agree).^17^ The total score corresponds to the sum of the 25 items, thus ranging from 25 to 175. A score below 121 is considered “low resilience,” 121 to 145 points “moderate resilience” and higher than 145 “high resilience.” The validated Portuguese version presented high internal consistency, with α = .89^18^ and α = .94 in a medical sample^19^ and α = .95 in our study.

DASS-21 is composed of 3 subscales evaluating depression, anxiety, and stress, each with seven 4-point Likert scales (0: did not apply to me at all; 1: applied to me sometimes; 2: applied to me often; 3: applied to me a lot or most of time).^20,21^ The depression subscale is scored as follows: normal (0-9), mild (10-13), moderate (14-20), severe (21-27), and extremely severe (28 or more). In the anxiety subscale, scores are as follows: normal (0-7), mild (8-9), moderate (10-14), severe (15-19), and extremely severe (20 or more). In the stress subscale, scores are as follows: normal (0-14), mild (15-18), moderate (19-25), severe (26-33), and extremely severe (34 or more). For DASS-21, the values of α in our study were .90, .84, and .90 for the depression, anxiety, and stress subscales, respectively. For the present analysis, each subscale was categorized into normal and not normal (including mild, moderate, severe, and extremely severe levels).

### Data Analysis

Data from the survey were exported to a Microsoft Excel^®^ 2016 (USA) spreadsheet and statistical analyses were performed using SPSS^®^ Statistics (version 26.0; SPSS Inc., Chicago, Illinois, USA).

Categorical variables were described using absolute and relative frequencies, n (%). Quantitative variables with a normal distribution were described with mean ($\bar{x}$), standard deviation (SD), minimum (min), and maximum (max) values. Those without a normal distribution were described using medians (med) and interquartile intervals [*Q*1; *Q*3] (in these cases, means and standard deviations were also included for comparison purposes). Normal distribution was assessed with visual inspection of the histograms.

The internal consistency of each subscale was assessed using Cronbach’s alpha (α), where a value higher than .7 was considered acceptable.^22^

Separate multiple logistic regressions were performed for personal, work-related, and patient-related burnout. The independent variables to include in each multiple logistic regression were chosen by conducting simple logistic regressions with each variable. All variables correlating with the outcomes with *P* ≤ .20 in the simple logistic regression were included in the multiple logistic regression analyses. Only the significant variables were maintained in the final multivariate models for each burnout dimension. To convey the results of logistic regressions odds ratios (OR), 95% confidence intervals [95% CI], and *P*-values are presented. The final models were evaluated using the Hosmer & Lemeshow test of adequate fit. *P* ≤ .05 was considered significant.

### Sample Size

Considering there are 8000 Portuguese physicians in a Primary Healthcare specialty (either Family Medicine or Public Health) we planned a sample size between 192 and 367 respondents, considering the most conservative scenario (a proportion of 50%), for a level of confidence of 95% and an error margin between 5% and 7%.

## Results

### Participants

225 primary care physicians answered the questionnaires, but 11 were excluded from the analysis for being either retired or absent from work due to disease or leave. Therefore, a total of 214 respondents were considered (80.8% female, mean age 38.6 years old). Of these 214 respondents, 95.3% were working on the frontline (defined as those indicating they worked face to face, full-time, or part-time) during the pandemic. The sociodemographic characteristics of the participants are described in Table 1.

**Table 1.** Sociodemographic Characteristics of Participants (n = 214).

Characteristics	
Sex, n (%)	
Female	173 (80.8)
Male	40 (18.7)
Non-binary	1 (0.5)
Age (years), $\bar{x}\pm SD$, min, max	38.6 ± 11.3, 24, 67
Marital status, n (%)	
Single	83 (38.8)
Married/de facto union	113 (52.8)
Divorced	18 (8.4)
Parental situation, n (%)	
No children	121 (56.5)
Have children	93 (43.5)
>12 years old	36 (38.7)
≤12 years old	57 (61.3)
Years of professional experience, n (%)	
≤5 years	71 (33.2)
6-15 years	85 (39.7)
≥15 years	58 (27.1)
Living situation during the COVID-19 outbreak, n (%)	
Living with friends	9 (4.2)
Living with family	164 (76.6)
Living alone	41 (19.2)
Changed their living situation during the pandemic, n (%)	39 (18.2)
Taking care of elderly people, n (%)	20 (9.3)
Living with a person at higher risk of complications from COVID-19, n (%)	70 (32.7)
Have any health condition, n (%)	72 (33.6)
Death of a relative or a friend during the pandemic, n (%)	7 (3.3)
Reduction in monthly income, n (%)	32 (15)
COVID-19 front line, n (%)	204 (95.3)
Working directly with infected people, n (%)	77 (36.0)
Feels they have adequate Personal Protection Equipment (n = 77), n (%)	48 (62.3)
Teleworking, n (%)	
Partial telework	10 (4.7)
In-person work only	194 (90.7)
Telework only	10 (4.7)
Area of residence (NUTS II), n (%)	
Norte	86 (40.2)
Centro	39 (18.2)
AM Lisboa	40 (18.7)
Alentejo	9 (4.2)
Algarve	6 (2.8)
Azores	8 (3.7)
Madeira	26 (12.1)
Sought help related with mental health, n (%)	24 (11.2)
Started a new drug related with mental health, n (%)	23 (10.7)
Have been in quarantine, n (%)	39 (18.2)
Got tested for COVID-19, n (%)	72 (33.7)
Diagnosed with COVID-19, n (%)	2 (0.9)

### Burnout Dimensions, Resilience, Depression, Anxiety, and Stress

**Figure 1.**
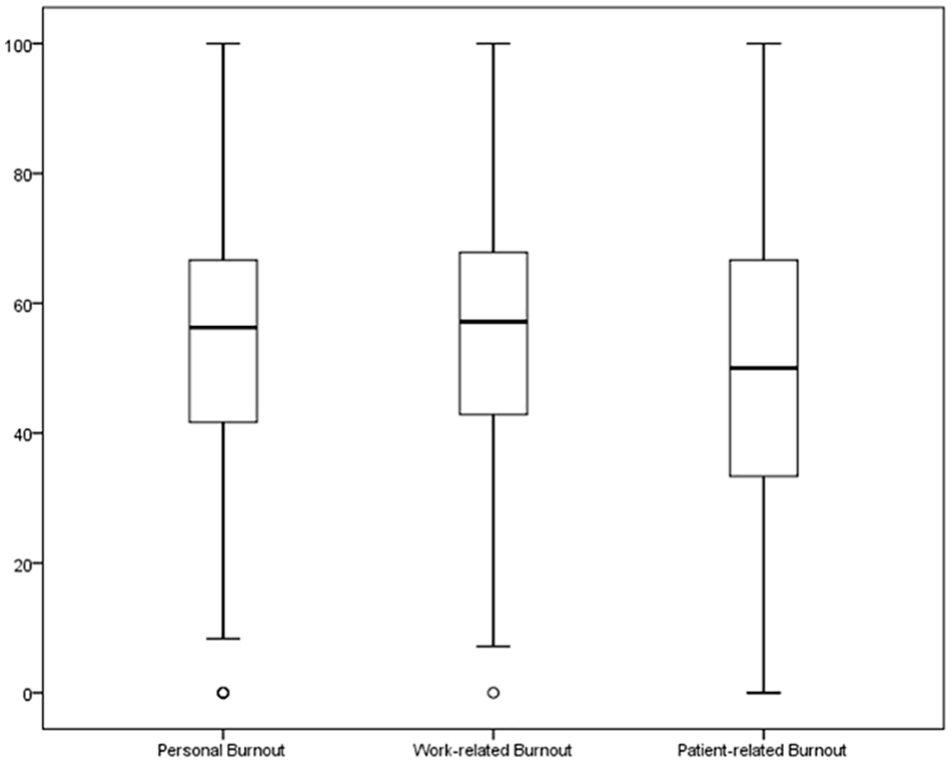
Boxplots for the 3 dimensions of burnout (n = 214).

High levels of burnout were found for the 3 dimensions: 65.9% for personal burnout, 68.7% for work-related burnout, and 54.7% for patient-related burnout (Figure 1 and Table 2). Resilience was high or moderate in 77.1% of the sample.

**Table 2.** Descriptive Statistics of Psychological Variables by Categories.

Variables	n (%)
Personal burnout (High level)	141 (65.9)
Work-related burnout (High level)	147 (68.7)
Patient-related burnout (High level)	117 (54.7)
Resilience	
Low	49 (22.9)
Moderate	109 (50.9)
High	56 (26.2)
Anxiety	
Normal	149 (69.6)
Mild	15 (7.0)
Moderate	28 (13.1)
Severe	11 (5.1)
Extremely severe	11 (5.1)
Stress	
Normal	132 (61.7)
Mild	29 (13.6)
Moderate	24 (11.2)
Severe	18 (8.4)
Extremely severe	11 (5.1)
Depression	
Normal	144 (67.3)
Mild	25 (11.7)
Moderate	21 (9.8)
Severe	14 (6.5)
Extremely severe	10 (4.7)

Participants reported levels of depression (67.3%), anxiety (69.6%), and stress (61.7%) which fell within the normal range on the measurement tool (Table 2).

### Logistic Regression Analysis

Results for the multiple logistic regression analyses for personal, work-related, and patient-related burnout are presented in Tables 3 to 5. Being a female was significantly associated with higher odds of patient-related burnout (OR = 2.57;95% CI = [1.17; 5.65]; *P* = .019). Having worked for 6 to15 years was also significantly associated with higher odds of patient-related burnout (OR = 3.12;95% CI = [1.53; 6.34]; *P* = .002) compared to those with 5 or fewer years of practice. A reduction in monthly income was significantly and inversely correlated with patient-related burnout.

**Table 3.** Multiple Logistic Regression Analysis with Personal Burnout as a Dependent Variable.

	Initial model		Final model	
	OR [95% CI]	*P*-value	OR [95% CI]	*P*-value
Resilience				
Low	*Reference*			
Moderate	0.66 [0.24; 1.81]	.417		
High	0.32 [0.1; 0.97]	.044		
Anxiety				
Normal	*Reference*		*Reference*	
Not normal	5.65 [1.67; 19.07]	.005	7.16 [2.36; 21.78]	.001
Depression				
Normal	*Reference*		*Reference*	
Not normal	2.5 [0.86; 7.22]	.091	4.76 [1.83; 12.4]	.001
Stress				
Normal	*Reference*			
Not normal	2.23 [0.87; 5.73]	.097		
Hosmer & Lemeshow test	$\chi_{HL}^{2}\left(5\right)=2.328;P=.802$		$\chi_{HL}^{2}\left(2\right)=0.215;P=.898$	

**Table 4.** Multiple Logistic Regression Analysis with Work-Related Burnout as a Dependent Variable.

	Initial model		Final model	
	OR [95% CI]	*P*-value	OR [95% CI]	*P*-value
Years of professional experience				
≤5 years	*Reference*		*Reference*	
6-15 years	2.57 [1.17; 5.62]	.018	2.41 [1.13; 5.17]	.023
≥15 years	0.94 [0.41; 2.13]	.876	0.85 [0.38; 1.89]	.683
Resilience				
Low	*Reference*			
Moderate	1.23 [0.46; 3.31]	.686		
High	0.61 [0.21; 1.79]	.365		
Anxiety				
Normal	*Reference*		*Reference*	
Not normal	3.59 [1.16; 11.09]	.026	4.18 [1.55; 11.23]	.005
Depression				
Normal	*Reference*		*Reference*	
Not normal	3.73 [1.13; 10.05]	.029	4.79 [1.82; 12.66]	.002
Stress				
Normal	*Reference*			
Not normal	1.74 [0.67; 4.52]	.257		
Hosmer & Lemeshow test	$\chi_{HL}^{2}\left(7\right)=7.477;P=.381$		$\chi_{HL}^{2}\left(5\right)=8.701;P=.122$	

**Table 5.** Multiple Logistic Regression Analysis with Patient-Related Burnout as a Dependent Variable.

	Initial model		Final model	
	OR [95% CI]	*P*-value	OR [95%CI]	*P*-value
Sex				
Masculine	*Reference*		*Reference*	
Feminine	2.53 [1.12; 5.70]	.025	2.57 [1.17; 5.65]	.019
Years of professional experience				
≤5 years	*Reference*		*Reference*	
6-15 years	3.34 [1.61; 6.93]	.001	3.12 [1.53; 6.34]	.002
≥15 years	0.82 [0.37; 1.83]	.632	0.80 [0.37; 1.75]	.576
Reduction in monthly income				
No	*Reference*		*Reference*	
Yes	0.43 [0.17; 1.09]	.076	0.36 [0.15; 0.88]	.025
Resilience				
Low	*Reference*			
Moderate	1.76 [0.73; 4.28]	.209		
High	1.12 [0.42; 3.02]	.823		
Anxiety				
Normal	*Reference*			
Not normal	1.28 [0.51; 3.19]	.600		
Depression				
Normal	*Reference*		*Reference*	
Not normal	2.34 [0.93; 5.85]	.070	3.33 [1.67; 6.64]	.001
Stress				
Normal	*Reference*			
Not normal	1.84 [0.78; 4.32]	.163		
Hosmer & Lemeshow test	$\chi_{HL}^{2}\left(8\right)=5.181;P=.738$		$\chi_{HL}^{2}\left(7\right)=7.820;P=.349$	

Higher levels of depression were significantly associated with higher levels of all 3 burnout dimensions: personal burnout (OR = 4.76; 95% CI = [1.83; 12.4]; *P* = .001), work-related burnout (OR = 4.79; 95% CI = [1.82; 12.66]; *P* = .002), and patient-related burnout (OR = 3.33; 95% CI = [1.67; 6.64]; *P* = .001), compared with normal levels.

Higher levels of anxiety were also significantly associated with higher levels of personal burnout (OR = 7.16; 95% CI = [2.36; 21.78]; *P* = .001) and work-related burnout (OR = 4.18; 95% CI = [1.55; 11.23]; *P* = .005).

Stress and resilience did not result in significant variables in the multiple model for the 3 dimensions of burnout.

The final models resulted in adequate fits to the observed values according to the Hosmer & Lemeshow test of fit results (Tables 3-5).

## Discussion

### Summary of Main Findings

This is to our knowledge the first study assessing burnout in primary care physicians during the ongoing COVID-19 pandemic. Our findings demonstrate a significant burden of burnout, anxiety, depression, and stress during these unprecedented times in the context of primary care. A strong association was found between gender, years of professional experience, depression and anxiety, and burnout levels.

Of note, an inverse correlation was found between patient-related burnout and reduction in monthly income; we hypothesize this may be due to less contact with patients and a lesser workload in the cases where a reduction in salary occurred.

### Comparison with Existing Literature

We found a higher prevalence of burnout compared to pre-COVID-19 levels, which is in line with other recent similar studies.^23^ In a 2016 Portuguese study of burnout among healthcare professionals, 43.6% of doctors were found to have high burnout, although comparison of this figure with our 55% to 69% prevalence rate should be made with caution, as the former study measured burnout with the Maslach Burnout Inventory.^3^

In a multinational, cross-sectional study of 3537 healthcare workers, 67% screened positive for burnout; gender, anxiety, and depression were significant determinants for burnout, in line with our results.^24^ A subgroup analysis in a systematic review and meta-analysis of depression, anxiety, and insomnia among healthcare workers during the COVID-19 pandemic revealed gender differences; according to our findings, female workers reported higher levels of affective symptoms.^25^ A 2008 study assessing burnout in family doctors found that approximately two thirds scored high for burnout in any dimension, although direct comparison with our findings is not possible due to the use of different scales.^26^

Another study assessing burnout during the pandemic found that younger age and being female were independent determinants of burnout, similar to our results.^27^ In Portugal, 6 to 15 years of experience in primary care coincides with the first years of practice as a specialist and, frequently, taking on a new role as parent; therefore, we can hypothesize that those factors contribute to the observed differences, although having children ≤12 years was not significantly associated with burnout in our study. Additionally, a previous Portuguese study found that healthcare professionals with more years on the job were less affected by burnout.^3^ Our findings are also consistent with the results of a study evaluating the prevalence of burnout and its associations with the work environment among hospital physicians in Lithuania, which used the CBI scale and found a significative inverse relationship between work- and patient-related burnout and length of employment and job control (assessed using the Job Content Questionnaire).^28^

### Strengths and Limitations

The use of the CBI scale to assess burnout is one of the strengths of our study: in addition to its excellent psychometric properties and validation for the Portuguese population, we found this scale to be particularly appropriate for the population being studied, since the CBI is composed of 3 different domains that also reflect primary care physicians’ daily activities.

Having used a convenience sample, our results may not be generalized to other populations and contexts. Additionally, we cannot rule out that the physicians who decided to answer our survey might be different from those who did not respond, nor can a self-reporting bias be excluded. Our sample consisted of 80.8% female physicians, which could suggest a gender-biased response, which may reflect the female predominance in family medicine in Portugal: in 2019, 62.2% of family doctors were female, with 73.2% females in the 36 to 40 years age group.^29^

The questionnaire may not address other important contributors to burnout. Notably, it is not possible to establish a causal relationship between the pandemic and our findings.

### Implications for Clinical Practice and Research

Burnout has negative impacts on physicians, patients, and healthcare organizations.^30^ Our findings reinforce that strategies to counteract physician burnout during a pandemic need to be further investigated. Our workgroup suggests next steps should include, at an organizational level, involving physicians in designing guidelines and contingency plans and also in implementing physician’s access to feedback channels. To feel better prepared, they should receive rapid basic training and have the opportunity to talk to experts. A supportive network should be created, including childcare, transportation, and lodging.^7^ Emotion management strategies and self-care should also be endorsed, encompassing rest, work breaks, sleep, shift work, fatigue, and healthy lifestyle behaviors.^31^ Physicians with depressive and anxious symptoms are at increased risk of personal and work-related burnout; therefore, specific interventions should be aimed at identifying and helping this group. Specific programs to prevent burnout should also be implemented for physicians just starting their careers, such as coping and self-care strategies for medical residents. More studies are needed in the long term to provide a comprehensive assessment of the impact of the COVID-19 pandemic and to further evaluate it as a contributing cause for burnout, for instance, in a study using a sequential mix-method approach.
